# 

**Published:** 1895-03

**Authors:** 


### CONTENTS:

### SELECTIONS.

Article I.—Dentists and the Census of 1890. Historical Sketch. By Richard Grady, M. D., D. D. S. - - - - 481

" II.—Facial Neuralgia, from A Dento-surgical Standpoint. By M. H. Cryer, M. D., D. D. S., - - - 495

" III.—Alveolar Abscess. By E. Herbert Adams, M. D., D. D. S - - - - - - - - - 509

" IV.—Filling Children's Teeth. By Dr. W. G. A. Bonwill.- 518

### EDITORIAL, ETC.

The Census of 1890. - - - - - - - 524

### MONTHLY SUMMARY.

Pulp-Treatment—Cap and Dressing. - - - - - 526

To Remove Plaster Impression from the Tray When it Hangs. - 527

Sensitive Dentine. - - - - - - - - 528

Diagnosis in Obscure Cases. - - - - - - 528

Cast Solid Gold Cusps. - - - - - - - 528

Pulp Capping in Deciduous Teeth. - - - - - 528

